# Psychological green climate as a mediator between green transformational leadership, innovation, and environmental awareness

**DOI:** 10.3389/fpsyg.2025.1701658

**Published:** 2025-11-20

**Authors:** Onur Oktaysoy, Ethem Topcuoglu, Murat Selim Selvi, Selen Uygungil-Erdogan, Yaşar Şahin, Volkan Tatar, Mahmut Özdemirkol, Engin Karafakioglu

**Affiliations:** 1 Faculty of Economics and Administrative Sciences, Kafkas University, Kars, Türkiye; 2 Academy of Civil Aviation, Giresun University, Giresun, Türkiye; 3 Faculty of Economics and Administrative Sciences, Tekirdağ Namik Kemal University, Tekirdağ, Türkiye; 4 Kadirli Faculty of Applied Sciences, Osmaniye Korkut Ata University, Osmaniye, Türkiye; 5 Beşikdüzü Vocational School, Trabzon University, Trabzon, Türkiye; 6 Faculty of Economics and Administrative Sciences, Istanbul Arel University, Istanbul, Türkiye; 7 Turkish Gendarmerie General Command, Ankara, Türkiye

**Keywords:** green transformational leadership, organizational green innovation, individual environmental awareness, psychological green climate, environmental psychology

## Abstract

**Introduction:**

The negative impacts of human activities on nature have brought about environmental problems such as global warming, water pollution, and deforestation. Mitigating these problems is closely related not only to environmental policies but also to internal leadership approaches within organizations. In this context, green transformational leadership (GTL) stands out as a contemporary leadership style that guides employees toward environmentally conscious behaviors and encourages sustainable practices. This study examines the effects of green transformational leadership on corporate green innovation and individual environmental awareness, aiming to reveal the mediating role of psychological green climate in this relationship from a Turkiye perspective.

**Methods:**

The study was structured as structural equation modeling (SEM). Research data were obtained from 435 Turkish SMEs employees selected using convenience sampling, and analyzed using the SmartPLS-SEM method.

**Results:**

The findings indicate that green transformational leadership encourages environmentally focused behaviors at both the organizational and individual levels, and that the psychological green climate plays a partial mediating role in these relationships. The findings show that green transformational leadership is a strategic tool in creating sustainable organizational cultures.

**Discussion:**

The study is innovative because it is one of the few in the literature that integrates organizational (green innovation) and individual (environmental awareness) outcomes within the same model, treating psychological green climate as an intermediary mechanism. In this context, the research offers practical recommendations to managers for strengthening the perception of green climate, encouraging environmentally friendly behaviors, and restructuring innovation processes from a sustainability perspective.

## 1 Introduction

As a result of the changes that have taken place in recent years, deforestation has accelerated, with 5.2 million hectares of forest lost annually between 2000 and 2010. The destruction of nature is causing ecosystem degradation, and it is estimated that 1.4 billion people are at risk of losing access to fresh water. Additionally, over 100 million people whose livelihoods depend on fishing are at risk due to the pollution of water sources, oceans, and seas (World Bank, 2023). These global environmental issues require not only governments but also businesses to redefine their environmental responsibilities. As a result, issues such as global climate change, depletion of natural resources, and environmental degradation have also been placed on the agenda of businesses (Rame et al., 2024). In today’s business world, environmental sustainability is not only an ethical obligation but also a strategic element that provides a competitive advantage (Fareed et al., 2023). At this point, leadership has become a key factor in embedding environmental sustainability goals into organizational culture (Din et al., 2025). GTL is considered an expanded version of transformational leadership theory from an environmental sustainability perspective (Özgül and Zehir, 2023). Based on Bass and Riggio's (2006) transformational leadership model, this type of leadership contributes to employees’ development of environmental awareness through the components of inspirational motivation, individualized consideration, intellectual stimulation, and idealized influence (Graves et al., 2019).

Green leaders encourage the development of sustainable behavior at both the individual and organizational levels by guiding their employees toward environmentally friendly practices within their organizations (Singh et al., 2020). Another critical component of sustainability in an organizational context is organizational green innovation. Green innovation refers to the adoption of innovative approaches in products, processes, and management systems by businesses to achieve environmental sustainability goals (Chen, 2008). According to research, environmentally friendly product innovations, energy-efficient production processes, and sustainable management practices enable companies to reduce their environmental impact and achieve long-term economic benefits (Horbach et al., 2012; Wang et al., 2022). Furthermore, it has been reported that investments in green technology and sustainable innovation increased by 8% annually worldwide as of 2022 (Xu et al., 2020). This suggests that environmental sustainability is becoming an increasingly significant concern for businesses. At the individual level, environmental awareness is defined by individuals’ levels of awareness of environmental issues and their tendency to adopt environmentally friendly behaviors (Stern, 2000). According to the Theory of Planned Behavior, individuals’ development of environmentally conscious behaviors is directly related to their attitudes, perceived behavioral control, and subjective norms. Environmentally conscious individuals contribute to environmental sustainability at both the individual and societal levels by developing sustainable consumption habits (Ayar and Gürbüz, 2021; Li et al., 2025). On the other hand, the concept of psychological green climate emerges as an essential variable in this process. Psychological green climate refers to employees’ perceptions of the norms, policies, and values established within the organization regarding environmental sustainability (Norton et al., 2015). Employees with a strong perception of psychological green climate contribute more to organizational sustainability practices and increase their environmental awareness levels (Chou, 2014).

While the existing literature has examined the relationships between GTL, psychological green climate, organizational green innovation, and individual environmental awareness separately, there is a lack of empirical evidence on how these factors interact. It is observed that studies examining these relationships comprehensively and testing the mediating effect of psychological green climate are limited, especially in developing countries (Liao et al., 2021; Rahmani et al., 2024; Öztürk et al., 2024; Han et al., 2025). Furthermore, industry-specific dynamics, cultural factors, and regulatory policies create significant differences in the adoption of green innovation processes (Le et al., 2024). This situation highlights the need for businesses to develop customized policies and practices that consider sectoral and regional dynamics, rather than adopting homogeneous approaches when determining their sustainability strategies (Demir et al., 2025).

This study aims to fill the aforementioned research gap by examining the effects of green transformational leadership on organizational green innovation and individual environmental awareness, within a comprehensive model framework mediated by the psychological green climate. Thus, the study aims to contribute theoretically and practically to a clearer understanding of leadership mechanisms that contribute to environmental sustainability at both the organizational and individual levels.

## 2 Conceptual framework

### 2.1 Green transformational leadership

GTL has emerged as an expanded version of transformational leadership theory, specifically in the context of environmental sustainability. It aims to encourage employees to adopt sustainable practices at the organizational level by promoting environmental awareness (Bass, 1985). While traditional transformational leadership asserts that visionary leaders motivate employees to achieve organizational goals (Bass and Avolio, 1994), GTL extends this effect to the field of environmental sustainability. Environmental sustainability is considered not only an ethical imperative in today’s business world but also a strategic element that provides a competitive advantage (Fareed et al., 2023). In this context, GTL promotes the development of sustainable behaviors at both the individual and organizational levels by raising employees’ environmental awareness (Din et al., 2025).

When examining the theoretical foundations of GTL, it is evident that it aligns with the core components of transformational leadership theory (Özdemirkol, 2020). Bass and Riggio (2006) address transformational leadership within the framework of four core components: inspirational motivation, individualized consideration, intellectual stimulation, and idealized influence. GTL integrates these components with an environmental sustainability perspective, enabling employees to develop environmental awareness and disseminate green practices within the organization (Graves et al., 2019). In particular, through idealized influence and inspirational motivation, leaders emphasize the importance of environmental sustainability to their employees and encourage them to take more initiative in this area (Singh et al., 2020).

When examining the effects of this leadership style, significant results emerge in terms of organizational innovation and environmental performance. In particular, the efforts of green leaders to develop environmental policies at the managerial level and encourage employees to implement these policies accelerate green innovation processes in businesses (Fareed et al., 2023). Green innovation enables businesses to reduce their environmental impact, thereby helping them meet regulatory pressures and gain a competitive advantage (Chen et al., 2023). Additionally, GTL has been found to support the development of individual environmental awareness by increasing employees’ perceptions of a green psychological climate (Norton et al., 2015). Employees’ perception of working in an environmentally conscious culture within the organization increases their environmentally friendly behaviors, thereby contributing to organizational sustainability efforts (Piwowar-Sulej et al., 2025).

### 2.2 Psychological green climate

Psychological green climate can be defined as a concept that expresses employees’ perceptions of the norms, policies, and values established within an organization regarding environmental sustainability (Norton et al., 2015). When evaluated within the organizational climate literature, psychological green climate is recognized as a crucial variable that explains how employees perceive the organization’s commitment to environmental sensitivity and how this perception influences their environmental attitudes and behaviors (Naz et al., 2023). In this context, psychological green climate is considered a critical factor that promotes both individual environmental awareness and sustainability practices at the organizational level (Akbar et al., 2024).

The concept of psychological green climate, based on organizational climate theory, examines how individuals perceive environmental sustainability policies within an organization and how this perception shapes their environmentally sensitive behavior (Biswas et al., 2021). The organizational climate theory developed by Schneider (1975) suggests that individuals’ shared perceptions of organizational processes guide their behavior in the workplace. When considered in a green context, psychological green climate promotes environmentally friendly organizational behavior by shaping employees’ perceptions of how much environmental sustainability is supported within the organization. Organizations’ explicit and consistent emphasis on their environmental sustainability commitments strengthens employees’ perceptions of psychological green climate, thereby facilitating the adoption of environmentally friendly business practices (Al-Romeedy et al., 2025).

One of the key factors supporting the development of a psychologically green climate is the clear and consistent implementation of organizational sustainability policies (Chen et al., 2014). Organizations’ adoption of green business practices sends strong signals to employees about their environmental responsibilities, thereby strengthening their perception of a psychological green climate (Dumont et al., 2017; Kadioglu et al., 2025). At this point, the implementation of sustainability-focused training programs by organizations is considered a crucial strategy for enhancing employees’ environmental awareness and fostering their commitment to environmentally friendly business practices (Boiral et al., 2015).

### 2.3 Organizational green innovation

Organizational green innovation is defined as the adoption of innovative approaches in products, processes, and management systems by businesses to achieve environmental sustainability goals (Chen, 2008). While traditional innovation focuses on technological and organizational developments to gain a competitive advantage and adapt to market conditions (Nguyen et al., 2025), green innovation integrates ecological and environmental factors into this process to support sustainable growth (Wang et al., 2022). Today, ecological sustainability is not only limited to legal regulations but is also considered a critical factor in helping businesses fulfill their social responsibilities and meet stakeholder expectations (Xu et al., 2020). In this regard, green innovation allows companies to minimize their environmental impact and gain a competitive advantage in the long run (Silvério et al., 2025).

Organizational green innovation is generally addressed in the literature within the framework of three basic dimensions: product innovation, process innovation, and management innovation (Chen et al., 2006). Green product innovation encompasses products developed using environmentally friendly materials and energy-efficient technologies (Dangelico and Pujari, 2010). Such innovations respond to consumers’ growing demand for sustainable products while also helping businesses reduce their carbon footprint (Li et al., 2022). Green process innovation is associated with the use of technologies that reduce waste, improve energy efficiency, and minimize environmental harm in production processes (Kouser et al., 2025). Businesses that optimize resource use and adopt sustainable production techniques gain advantages in terms of both operational efficiency and compliance with environmental regulations (Fu et al., 2025). Green management innovation, on the other hand, focuses on the development of environmentally friendly business models, the adoption of sustainable strategies, and the alignment of organizational culture with environmental values (Rietze et al., 2025b). Management innovations ensure the widespread adoption of green business strategies throughout the organization while also enabling the effective implementation of organizational sustainability policies (Zeng et al., 2022).

On the other hand, organizational green innovation contributes significantly not only to ensuring environmental sustainability but also to enhancing market competitiveness (Özdemirkol, 2024). When considered together with GTL, leaders’ motivation of employees in terms of environmental awareness and sustainability accelerates green innovation processes (Singh et al., 2020). Leaders who enhance employees’ environmental awareness contribute to the development of a sustainable innovation culture within the organization, thereby creating a long-term competitive advantage (Sun et al., 2025).

### 2.4 Individual environmental awareness

Individual environmental awareness is considered a multidimensional concept that encompasses the level of awareness, knowledge, and environmentally friendly attitudes that individuals have regarding environmental issues, sustainability, and their impact on ecosystems. This concept is evaluated across a broad spectrum, ranging from individuals’ perception of their ethical responsibilities toward the environment to their sensitivity to environmental issues and their demonstration of sustainable behaviors (Wallnoefer and Riefler, 2022). Today, raising individual environmental awareness is a critical issue in preventing large-scale environmental threats such as global warming, depletion of natural resources, and environmental pollution (Saifulina et al., 2022). Environmentally conscious individuals make environmentally sensitive decisions at both the individual and societal levels by developing sustainable consumption habits (Bouzari et al., 2022).

When examining the fundamental factors that influence the development of individual environmental awareness, it is evident that environmental education, social norms, and motivation based on personal values are crucial (Kollmuss and Agyeman, 2002). Environmental education programs help individuals understand environmental issues, thereby increasing their level of ecological awareness (Husin et al., 2025). Social norms, on the other hand, emerge as external factors that shape individuals’ environmental attitudes and encourage them to behave in an environmentally responsible manner (Niu et al., 2023). Research indicates that an individual’s environmental awareness is influenced not only by personal values but also by social norms and structural factors, such as environmental policies (Stern, 2000). When examining the relationship between individual environmental awareness and organizational sustainability, it is seen that employees’ environmental awareness plays a critical role in the success of green policies within the organization (Sánchez-García et al., 2025). In particular, GTL significantly impacts the development of employees’ individual environmental awareness, and leaders’ emphasis on environmental values enhances employees’ orientation toward sustainable behavior (Iosifidi, 2016). In this context, organizations can develop education and incentive mechanisms to enhance environmental awareness levels, thereby strengthening the alignment between individual environmental awareness and organizational sustainability goals (Oktaysoy et al., 2025).

## 3 Theoretical framework and hypotheses

Green transformational leadership has a strong relationship with stakeholder theory (Freeman, 2010). This theory, which argues that organizations should consider not only their shareholders but all stakeholders in the context of expectations, makes environmental sustainability an integral part of modern business management (Odabaş, 2024). Green transformational leaders strive to optimize not only the financial success of the organization but also its social and environmental impacts by directing employees toward environmentally friendly practices (Ledi et al., 2024). In this context, green transformational leaders also encourage the adoption of green innovation and sustainability strategies in a way that creates benefits for both individuals and the organization (Pham et al., 2022).

Green innovation provides the organization with a competitive advantage through the development of environmentally friendly new products and processes. Based on this, it is possible to say that organizational green innovation is influenced by the Resource-Based View (RBV) (Barney, 1991) and Dynamic Capabilities Theory (Teece, 2007). RBV argues that businesses must possess rare, inimitable, and value-creating resources to achieve sustainable competitive advantage (Hart, 1995). Green innovation enables businesses to develop such strategic resources by investing in environmentally friendly technologies, sustainable production processes, and ecological knowledge (Chen et al., 2006). The theory of dynamic capabilities, on the other hand, suggests that businesses must have the ability to manage learning, transformation, and innovation processes to adapt quickly to changing environmental and market conditions (Teece, 2007). In this context, green innovation enables businesses to increase their strategic flexibility by considering environmental factors and to evaluate opportunities for sustainable growth (Rietze et al., 2025a).

Individual environmental awareness is considered a critical variable in the formation of environmentally conscious individual behaviors. It encompasses various components such as a sense of environmental responsibility, ecological awareness, and sustainable consumption behaviors (Stein et al., 2025). Environmental responsibility refers to individuals’ awareness of their impact on ecosystems and their desire to minimize this impact (Turan-Torun et al., 2025). Schwartz (1977)'s Norm Activation Model argues that individuals shape their environmental behaviors based on ethical and moral norms. According to this model, individuals’ environmental sensitivity is triggered by the activation of their personal moral norms and their awareness of environmental issues (Park and Ha, 2014).

Based on the theories mentioned above, green transformational leadership encourages the sharing of environmental norms within the organization by setting an example for employees with its environmentally conscious vision and ethical sensitivity. This emerging shared perception forms the basis of the psychological green climate that determines how employees perceive the organization’s environmental policies. A framework has been outlined, suggesting that employees may be more inclined to exhibit environmentally conscious behaviors in response to leaders who value environmental sustainability. Such a climate can contribute to increased environmental awareness at the individual level while strengthening corporate green innovation at the organizational level. Therefore, it is predicted that the psychological green climate may serve as a fundamental mechanism, facilitating the social and cognitive processes from green transformational leadership to both organizational innovation and individual environmental awareness.

The relationship pattern between the concepts has been clarified below, and as a result, the hypotheses of the study have been developed.

### 3.1 The effect of green transformational leadership on psychological green climate

The relationship between GTL and psychological green climate is explored in the literature, particularly regarding how leaders’ guidance and value-oriented approaches to environmental sustainability influence employees’ perceptions of green practices (Robertson and Barling, 2013). GTL enhances employees’ perceptions of the psychological green climate by fostering environmentally friendly norms and policies within the organization and increasing their awareness of environmental sustainability (Norton et al., 2015). Leaders’ integration of environmental values into organizational processes and their encouragement of employees to participate in environmentally friendly practices contribute to the development of a psychological green climate by creating a shared sense of sustainability within the organization (Kim et al., 2020). Employees guided by leaders who support green policies within the organization feel a stronger sense of responsibility toward the environment. They are more likely to actively participate in organizational sustainability efforts (Graves et al., 2019). Previous research shows that GTL has a positive influence on employees’ environmental perceptions and the alignment of the organizational climate with green norms (Singh et al., 2020). In particular, leaders’ provision of inspirational motivation and guidance to employees on environmental sustainability leads to the strengthening of psychological green climate within the organization and the spread of environmentally friendly organizational behaviors (Chou, 2014). In this context, GTL is considered a fundamental element shaping employees’ perceptions of environmental sustainability within the organization. Based on this relationship pattern, the first hypothesis of the study, H1, was formulated.

*H1*: GTL has a positive and significant effect on psychological green climate.

### 3.2 The impact of psychological green climate on organizational green innovation

In organizations where a psychological green climate prevails, employees are encouraged to take on more environmental responsibilities and participate in green innovation processes (Kim et al., 2020). A strong emphasis on environmental sustainability within the organization encourages employees to develop innovative and environmentally friendly ideas, engage more in green business practices, and enhance the organization’s capacity for environmental innovation (Singh et al., 2020). Research in the literature indicates that a psychological green climate fosters the adoption of green innovation at the organizational level by enhancing employees’ commitment and motivation toward environmental innovation processes within the organization (Robertson and Barling, 2013). When employees’ perceptions of the organization’s policies promoting environmental sustainability are strengthened, they contribute more to green innovation processes (Graves et al., 2019). A strong psychological green climate within an organization enables the promotion of environmental innovations, allowing employees to be more creative in developing sustainable products, processes, and services (Chen et al., 2014). Based on the aforementioned relationship pattern, the second hypothesis of the study, H2, was formulated.

*H2*: Psychological green climate has a positive and significant effect on organizational green innovation.

### 3.3 The impact of green transformational leadership on organizational green innovation

The relationship between GTL and organizational green innovation is often addressed in the literature in terms of leaders’ visions that promote environmental sustainability and their ability to direct employees toward green innovation processes (Chen et al., 2014). This leadership style enhances employees’ environmental awareness while also cultivating a culture that promotes the development of sustainable business processes within the organization (Singh et al., 2020). Green innovation is recognized as a crucial factor in promoting sustainable growth, enabling businesses to develop environmentally friendly products and processes (Chen et al., 2006). In particular, leaders’ inspiring motivation and intellectual encouragement regarding environmental sustainability help employees contribute more to green innovation processes and generate creative solutions for environmental improvements (Graves et al., 2019). Research indicates that the presence of GTL in organizations significantly supports the adoption of green innovation practices at the organizational level by increasing employees’ environmental awareness (Robertson and Barling, 2013). Additionally, this leadership style facilitates organizations’ compliance with environmental regulations and contributes to their increased market competitiveness (Wu et al., 2021). Based on this relationship pattern, the third hypothesis of the study, H3, is formulated below.

*H3*: GTL has a positive and significant effect on organizational green innovation.

### 3.4 The effect of psychological green climate on individual environmental awareness

In organizations with a high psychological green climate, employees tend to develop environmentally friendly attitudes and behaviors at the individual level by showing greater sensitivity to environmental issues (Kim et al., 2020). When employees feel that the organization supports environmental sustainability policies and encourages green practices, this increases their awareness of environmental issues and helps them internalize environmentally friendly behaviors (Chou, 2014). When there is a strong emphasis on sustainability within an organization, employees are more likely to make environmentally conscious decisions not only at work but also in their daily lives (Paillé and Boiral, 2013). Research in the literature suggests that a psychological green climate fosters environmental awareness in individuals, enabling them to develop sustainable consumption habits and adopt environmentally friendly lifestyles (Boiral et al., 2015). In organizations that foster environmental responsibility within their culture, an atmosphere is created that supports the development of individual environmental awareness among employees, leading them to become more conscious and responsible in environmental matters (Graves et al., 2019). In this context, psychological green climate is considered a crucial organizational factor that fosters the development of individual environmental awareness by helping employees become more aware of environmental sustainability issues. Based on this relationship pattern, the fourth hypothesis of the study, H4, was formulated.

*H4*: Psychological green climate has a positive and significant effect on individual environmental awareness.

### 3.5 The impact of green transformational leadership on individual environmental awareness

GTL encourages its employees to become more environmentally conscious and contribute to the development of individual environmental awareness through its vision and exemplary behavior that promotes environmental sustainability (Graves et al., 2019). The inspiring motivation and intellectual encouragement provided by transformational leaders increase employees’ awareness of environmental issues, helping them become more conscious and responsible individuals (Kim et al., 2020). Additionally, leaders’ attitudes toward environmental sustainability serve as a model for employees, strengthening their environmentally friendly behaviors (Singh et al., 2020). In particular, the guidance of GTL to their employees on environmental responsibilities helps them internalize environmentally friendly behaviors, thereby increasing sustainability awareness at the individual level (Norton et al., 2015). Employees’ adoption of environmentally friendly behaviors facilitates sustainable practices not only within the organization but also in their daily lives (Boiral et al., 2015). Previous research shows that green leadership has a direct impact on the development of environmental awareness at the individual level, as it increases employees’ environmental awareness (Paillé and Boiral, 2013). Based on the relationship pattern in question, the fifth hypothesis of the study, H5, was formulated.

*H5*: GTL has a positive and significant effect on individual environmental awareness.

Furthermore, these conceptual relationships suggest that green transformational leadership is a style that encourages employees’ environmentally conscious attitudes and behaviors (Iqbal et al., 2023). This leadership style contributes to the formation of a psychological green climate by shaping the way environmental values, policies, and norms are perceived within the organization. The psychological green climate is a collective perception system that reflects how employees perceive their organization’s environmental sensitivity. This perception can have both organizational and individual-level consequences (Liu and Yu, 2023). In this context, the effects of GTL on organizational green innovation and individual environmental awareness can be direct or indirect, depending on the psychological green climate. At the organizational level, a perception of a green climate can strengthen organizational green innovation by encouraging the sharing of innovative ideas, the adoption of environmentally friendly practices, and process innovations. At the individual level, it can increase employees’ commitment to environmental values and raise their level of individual environmental awareness. Based on this prediction, hypotheses H6 and H7 are formulated below.

*H6*: Psychological green climate mediates the effect of GTL on organizational green innovation.

*H7*: Psychological green climate mediates the effect of GTL on individual environmental awareness.

**Figure 1:**
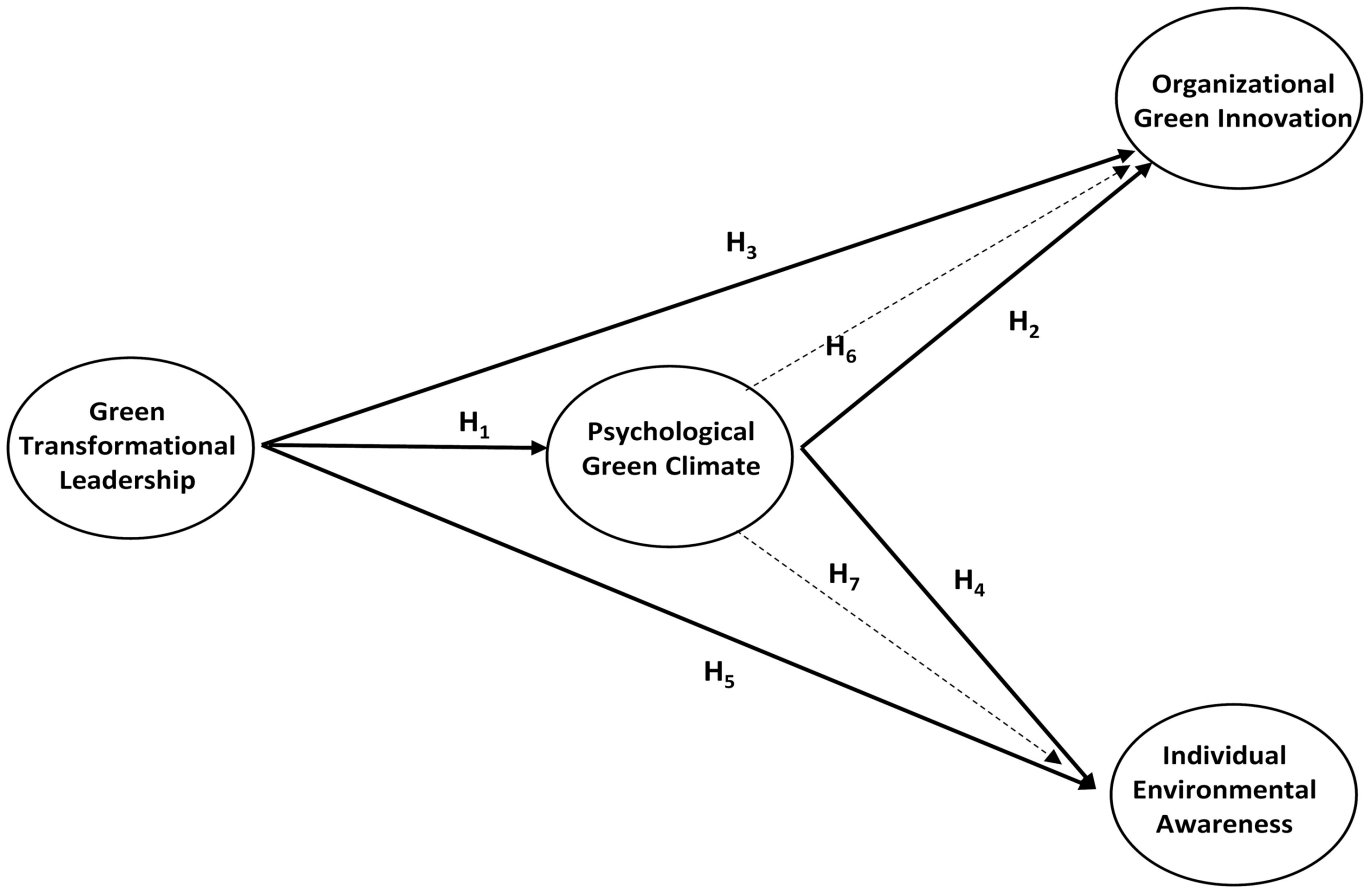
Research model.

Figure 1 shows the hypotheses developed in the scope of the research.

## 4 Method

### 4.1 Procedures and data

The purpose of this study is to determine the mediating role of psychological green climate in the effect of GTL on organizational green innovation and individual environmental awareness. To ensure compliance with scientific and ethical requirements, research approval was obtained from the Ethics Committee of Giresun University on May 9, 2025, with decision number 05/182. In this research, which is based on quantitative research methods, the influence and mediation relationships between the concepts under consideration were revealed through statistical analyses using SmartPLS-SEM. The reason for preferring SmartPLS over covariance-based SEM (CB-SEM; AMOS, LISREL) models is that the scales contain multidimensional and mixed reflective–formative structures, and the PLS-SEM method is more suitable for analyzing such complex models (Hair et al., 2019). Additionally, SmartPLS-SEM is a frequently preferred method due to its ability to simultaneously estimate multiple and dependent relationships between variables and measure latent structures simultaneously. SmartPLS-SEM is a frequently preferred method due to its ability to simultaneously estimate multiple and dependent relationships between variables and measure latent structures simultaneously. The research population comprises employees of small and medium-sized enterprises (SMEs) located in the provinces of Istanbul, Kars, and Giresun. Although the population size is not precisely known, it is estimated that the sample size will be at least 1,000,000 individuals. It has been calculated that a sample size of at least 385 individuals is required to achieve a 95% confidence interval for the representativeness of the sample (Cochran, 1977). The research data were collected using the convenience sampling method with the assistance of questionnaire forms. A 5-point Likert scale was used in all questionnaires, with responses ranging from 1 = “Strongly Disagree” to 5 = “Strongly Agree.” The forms were distributed to participants using the convenience sampling technique. The data was collected between May 10, 2025, and July 26, 2025. With the number of acceptable data points reaching 435, the sample size was deemed sufficient, and data collection was terminated, allowing for the analysis phase to proceed.

The study was first written in Turkish and reviewed by the authors. It was then translated into English, reviewed again, and proofread using Grammarly Pro to ensure accuracy.

### 4.2 Scales for variables

In the study, the scale developed by Chen and Chang (2013) and adapted into Turkish by Kerse et al. (2021) was used to measure GTL. The scale consists of six questions and a single dimension, and its reliability (Cronbach’s Alpha: 0.903) was found to be high by Chen and Chang (2013).

To measure psychological green climate, the Green Psychological Climate Scale, developed by Norton et al. (2014) and adapted into Turkish by Erbaşı (2021), was used. The scale consists of 5 items and a single factor, and its reliability (Cronbach’s Alpha: 0.920) was found to be high in the study conducted by Erbaşı (2021).

To measure individual environmental awareness, a 4-item, single-dimensional scale developed by Ding et al. (2023) was used in the study as another variable. In the study, the scale’s reliability, as measured by Cronbach’s Alpha (0.922), was found to be high.

The final scale used in the study is the Organizational Green Innovation Scale. The scale developed by Li et al. (2022) consists of five items and a single dimension; the study found that the scale was reliable (Cronbach’s Alpha = 0.776).

## 5 Findings

The study involved 435 participants, and information about them is presented in Table 1. Upon examination of the table, it is evident that the number of male participants (71.50%) is higher, and the number of married participants (69.70%) is significantly higher than that of single participants. The majority of participants hold a bachelor’s degree (63.90%), and the workforce is predominantly young, with the majority of employees falling within the 31–40 age range (54.90%). Additionally, it was found that the majority of participants have 10 years or less of professional experience (52.50%).

**Table 1:** Demographic findings related to participants.

Demographic	Variable	*n*	%
Gender	Female	124	28.50
	Male	311	71.50
Marital Status	Married	303	69.70
	Single	132	30.30
Age	Between 18 and 30 years old	82	18.90
	Between 31 and 40 years old	239	54.90
	Between 41 and 50 years old	91	20.90
	51 years old and above	23	5.30
Education	High school	53	12.20
	Associate degree	278	63.90
	Bachelor’s degree	98	22.50
	Postgraduate	6	1.40
Experience	5 years and under	62	14.30
	Between 6 and 10 years	166	38.20
	Between 11 and 15 years	96	22.00
	Between 16 and 20 years	77	17.70
	21 years and over	34	7.80
Income	Between 40.000 and 50.000 Turkish Lira	64	14.70
	Between 50.001 and 60.000 Turkish Lira	143	32.90
	Between 60.001 and 70.000 Turkish Lira	103	23.70
	Between 70.001 and 80.000 Turkish Lira	91	20.90
	80.001 Turkish Lira and above	34	7.80

Certain values are crucial for the reliability and validity of the data obtained from the study. At this point, factor load values must be above 0.50, Cronbach’s Alpha (CA), Composite Reliability (CR), and rho_A values must be above 0.70, and Average Variance Extracted (AVE) values must be above 0.50 (Sarstedt et al., 2022). When examining the values related to the scales and presented in Table 2, it is observed that all structures are above the acceptable threshold values, thus ensuring scale internal consistency and convergent validity (Sagbas et al., 2023).

**Table 2:** Factor load values, validity and reliability.

Item	Factor loading	Median	Standard deviation	Kurtosis	Skewness
Green transformational leadership					
CA = 0.840, rho_A = 0.839, CR = 0.882, AVE = 0.556					
GTL1	0.658	3.011	1.049	−0.716	−0.119
GTL2	0.702	3.161	1.042	−0.604	−0.118
GTL3	0.729	3.264	0.955	0.003	−0.407
GTL4	0.778	2.743	1.012	−0.402	0.226
GTL5	0.804	2.736	1.071	−0.696	0.170
GTL6	0.794	2.745	1.047	−0.609	0.151
Psychological green climate					
CA = 0.820, rho_A = 0.823, CR = 0.876, AVE = 0.588					
PGC1	0.768	3.547	0.927	0.605	−0.859
PGC2	0.768	3.538	0.902	0.543	−0.839
PGC3	0.605	2.487	0.872	−0.003	0.008
PGC4	0.859	3.299	0.965	−0.196	−0.521
PGC5	0.809	3.315	0.947	−0.330	−0.519
Organizational green innovation					
CA = 0.864, rho_A = 0.865, CR = 0.902, AVE = 0.647					
OGI1	0.828	2.995	0.964	−0.352	−0.316
OGI2	0.782	3.230	1.032	−0.452	−0.497
OGI3	0.803	2.917	1.045	−0.630	−0.149
OGI4	0.797	2.869	1.026	−0.453	0.098
OGI5	0.811	2.680	1.004	−0.276	0.332
Individual environmental awareness					
CA = 0.821, rho_A = 0.826, CR = 0.881, AVE = 0.650					
IEA1	0.770	2.628	0.956	−0.318	0.108
IEA2	0.799	2.772	0.969	−0.380	0.074
IEA3	0.853	2.848	0.957	−0.423	−0.025
IEA4	0.801	2.480	0.995	−0.395	0.328

CA = Cronbach’s Alpha CR = Composite Reliability, AVE = Average Variance Extracted.

One of the most commonly used methods for assessing reliability and consistency in research models is the CA value. The analysis revealed that all scales had CA values above 0.70, confirming that the scales were reliable and consistent. However, since CA values are greatly influenced by the number of variables and sample size, there is a view in the literature that measurement using CR would be more robust (Hair et al., 2019). Table 2 shows that all CR values are above 0.70, indicating that reliability and consistency are ensured. Furthermore, the fact that the AVE value is above 0.50 and the CR value is higher than the AVE value indicates that convergent validity is ensured (Sarstedt et al., 2022).

The CR and AVE values calculated based on the model are directly related to the factor loading value. The high value of this factor, which explains the relationship of the variable with the entire scale, provides important information regarding the reliability, validity, and consistency of the scale. In the literature, factor loading values are often desired to be above 0.50. All scale items obtained in the study have values above 0.50. Based on this, it can be said that the factor loadings indicate a strong scale structure (Widaman and Helm, 2023).

Since the variance inflation factor (VIF) values of the scale statements used in the study were below 10, it was determined that there was no common method bias for the statements (O’brien, 2007). The findings obtained from the analyses, when evaluated in the context of threshold values, indicate that the scales do not exhibit common method bias or multicollinearity (Hair et al., 2017).

The findings from the validity, reliability, and common method bias analyses indicate that the scales are insufficient in terms of scientific adequacy within the scope of the research. Additionally, a discrimination validity analysis is necessary to ensure that the scales can be distinguished from one another (Hair et al., 2017). Discriminant validity is a measure used to determine the extent to which variables in a scale are distinguished from other scale items (Sarstedt et al., 2022). The most frequently preferred methods in the literature for determining discriminant validity are the criteria proposed by Fornell and Larcker (1981) and Henseler et al. (2015). Therefore, in this study, the discriminant validity values, as per the Fornell and Larcker and Heterotrait-Monotrait criteria, are presented in Table 3.

**Table 3:** Discriminant validity.

Items	Fornell-Larcker Criterion and Heterotrait-Monotrait Ratio (HTMT)			
	1	2	3	4
GTL	0.746	0.726*	0.751*	0.702*
Individual environmental awareness	0.616	0.806	0.769*	0.726*
Organizational green innovation	0.651	0.649	0.804	0.738*
Psychological green climate	0.599	0.603	0.624	0.767

Values marked with * belong to HTMT analysis.

Analyses conducted to assess construct validity are crucial for determining whether there is a high degree of overlap among the model’s measurement variables and for evaluating the adequacy of inter-construct differentiation (Uygungil-Erdogan et al., 2025). In the Fornell-Larcker Criterion, the square roots of the AVE coefficients are used to ensure construct validity (Hair et al., 2017). According to the Heterotrait-Monotrait criteria, the relatedness threshold value must be below 0.90. The values obtained in this study met the threshold requirements for all variables, indicating that each scale structure is distinct and separate (Henseler et al., 2015).

**Figure 2:**
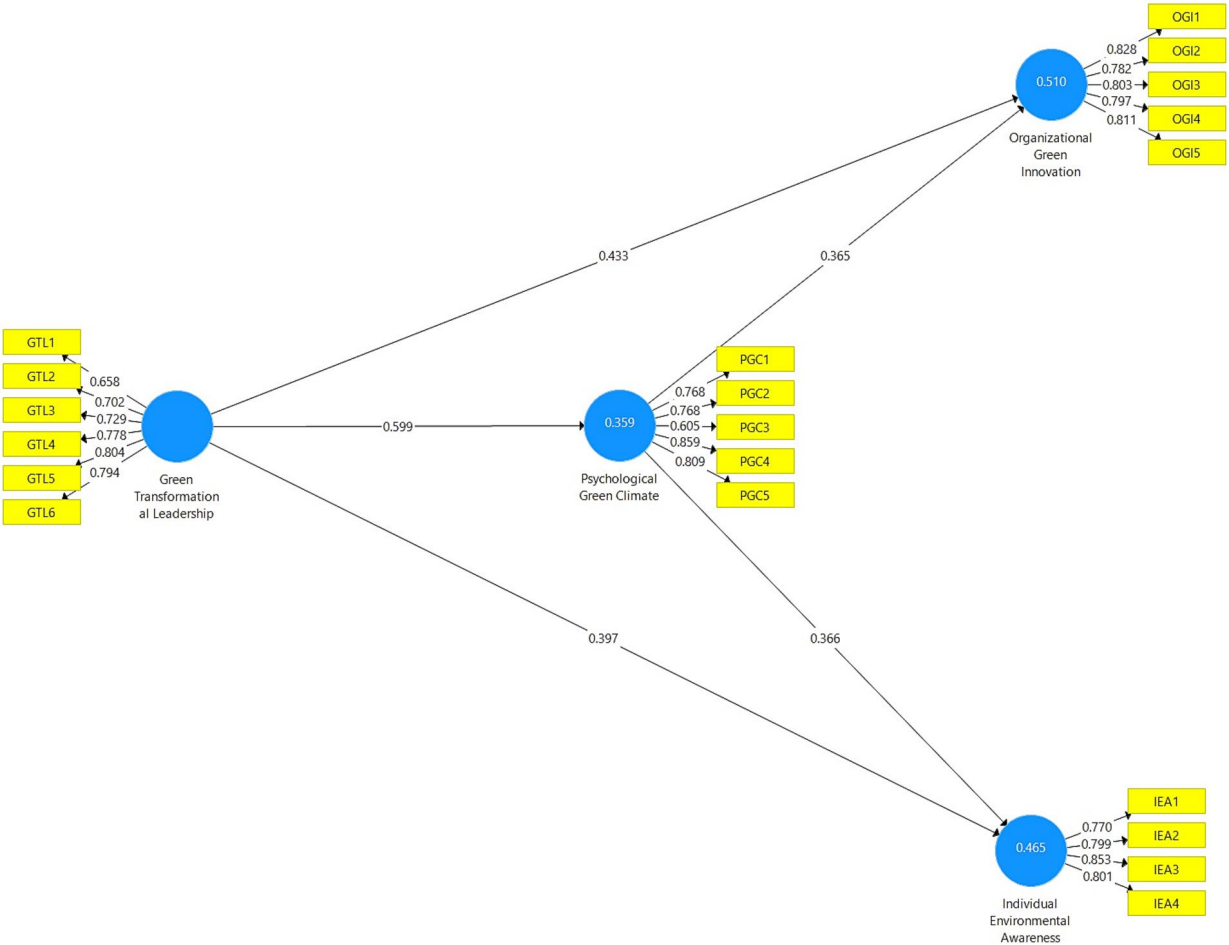
Path diagram.

To test the model, a mediation analysis was conducted using the Smart-PLS analysis application with a bootstrapping sample size of 5.000. In the bootstrap test, the sample size of 435 in the study is randomly increased to 5.000 using the Smart-PLS program and analyzed based on this number (Hair et al., 2017). The bootstrapping method assists in data analysis by accurately estimating standard errors and confidence intervals for path coefficients (Efron, 1979; Turan-Torun et al., 2025). On the other hand, this method also provides a reliable basis for hypothesis testing (Hair et al., 2017). As a result of the test, beta, p, and t values were examined to determine whether the path coefficients were statistically significant. The Smart-PLS diagram, obtained from the research model presented in Figure 2.

Figure 2 presents the structural model of the study. When examining the goodness-of-fit values obtained from the model, SRMR<0.080, d_ULS value 1.561, and d_G value 0.381 were determined. Additionally, the Chi-Square value was found to be 0.896, and the NFI value was 0.807. These values obtained from the model indicate that the model fit values are within acceptable limits. Looking at the relevant values, it is recommended that the Standardized Root Mean Square Residual (SRMR) value be below 0.08 and the Normalized Fit Index (NFI) value be above 0.80 (Byrne, 2016; Turan-Torun et al., 2025). Since the obtained results exceed the threshold values, the model meets the goodness-of-fit criteria, and hypothesis tests were performed (Turan-Torun et al., 2025). The values related to the hypothesis tests are presented in Table 4.

**Table 4:** Hypothesis test result.

Path analysis	Estimate	Standard deviation	t-values	*p*	Support
GTL → Psychological Green Climate	0.599	0.034	17.675	0.000	H1 Accept
Psychological Green Climate → Organizational Green Innovation	0.365	0.046	7.956	0.000	H2 Accept
GTL → Organizational Green Innovation	0.433	0.047	9.204	0.000	H3 Accept
Psychological Green Climate → Individual Environmental Awareness	0.366	0.045	8.038	0.000	H4 Accept
GTL - > Individual Environmental Awareness	0.397	0.045	8.736	0.000	H5 Accept
GTL → Psychological Green Climate → Organizational Green Innovation	0.219	0.032	6.743	0.000	H6 Accept Partial
GTL → Psychological Green Climate → Individual Environmental Awareness	0.218	0.032	6.887	0.000	H7 Accept Partial

**Figure 3:**
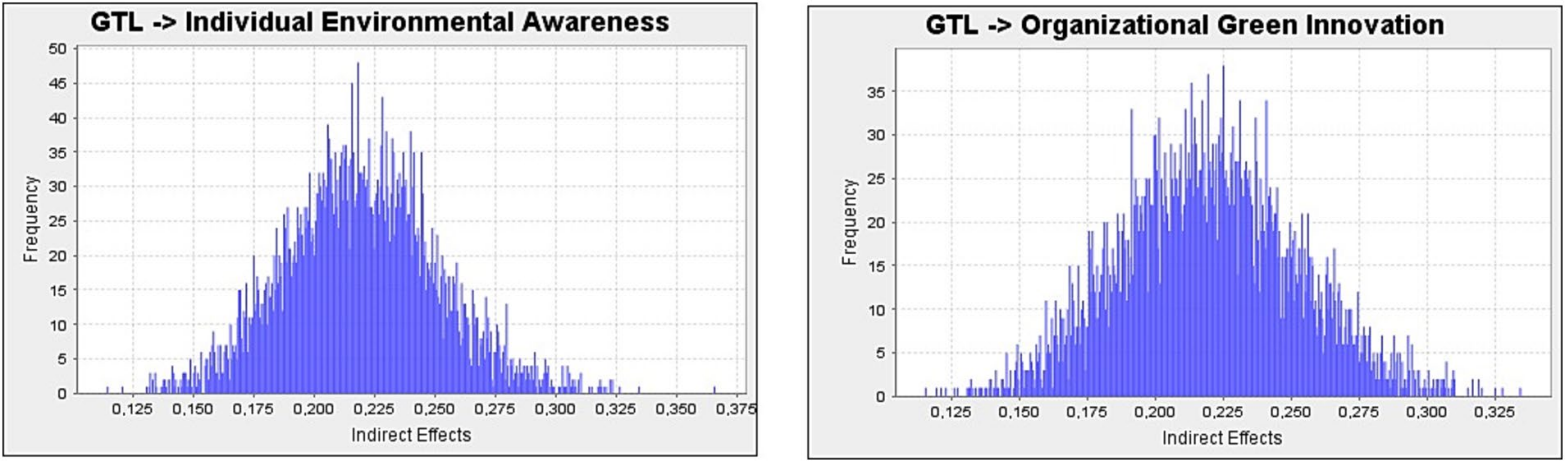
Mediation analysis diagrams.

The results of the analyses revealed that GTL had a significant and positive effect on the psychological green climate (*β* = 0.599, *p* < 0.01), and in this context, the H1 hypothesis was accepted. Additionally, the analyses revealed that the psychological green climate has a significant and positive effect on organizational green innovation (*β* = 0.365, *p* < 0.01), supporting the acceptance of H2. The effect of GTL on organizational green innovation (*β* = 0.433, *p* < 0.01) was found to be significant and positive, and therefore, the H3 hypothesis was accepted. Analyses conducted to test the fourth hypothesis of the study revealed that a psychological green climate has a significant and positive effect on individual environmental awareness (*β* = 0.366, *p* < 0.01). Therefore, the H4 hypothesis was accepted. Tests conducted in the context of the fifth hypothesis of the study revealed that GTL has a significant and positive effect on individual environmental awareness (*β* = 0.397, *p* < 0.05); therefore, the H5 hypothesis was accepted. In the mediation analyses conducted to determine the role of psychological green climate in the inter-conceptual relationship, it was found that psychological green climate plays a mediating role (*β* = 0.219, *p* < 0.01) in the effect of GTL on organizational green innovation, and the H6 hypothesis was accepted. Similarly, it was determined that psychological green climate plays a mediating role (*β* = 0.218, *p* < 0.01) in the effect of GTL on individual environmental awareness, and the H7 hypothesis was also accepted. For a better understanding of the mediation effect, a mediation diagram with effect sizes is presented in Figure 3.

With the acceptance of the hypotheses regarding the mediating role of psychological green climate in the relationships between the concepts under consideration, it is necessary to examine the significance of the hypotheses on the Variance Accounted for (VAF) value to determine the level of mediation (Hair et al., 2017). In this context, the calculations performed to determine the VAF value indicate that a value of 0–20% indicates no mediation, 20–80% indicates partial mediation, and 80–100% indicates full mediation (Henseler et al., 2015). The VAF value calculations are presented in Table 5.

**Table 5:** VAF values.

VAF value for H6				VAF value for H7			
a	b	c^l^	Result	a	b	c^l^	Result
0.599	0.365	0.433		0.599	0.366	0.397	
	A*B	(a*b) + C			A*B	(a*b) + C	
	0.218	0.651	%34		0.219	0.616	%36

^l^This sign is used to explain that the effect value is obtained after the mediation effect.

When examining the VAF calculations in Table 5, it was determined that neither of the H6 and H7 hypotheses, which were considered to have a mediating relationship, showed direct mediation in terms of VAF value, but rather partial mediation (20–80%) (Uygungil-Erdogan et al., 2025). Q^2^ analysis is expected to be performed to determine the quality of the analyses conducted in the structural equation modeling. The Q^2^ value must be above zero (Hair et al., 2017). The findings of the analysis conducted in this context are presented in Table 6.

**Table 6:** *R*^2^ test result.

Latent variable	*R* ^2^	R^2^ Adj.	Q^2^
Individual environmental awareness	0.465	0.462	0.298
Organizational green innovation	0.510	0.507	0.326
Psychological green climate	0.359	0.357	0.203

R^2^ is a statistical measure that indicates the proportion of variance in the dependent variable explained by the independent variables. Q^2^ is a coefficient that indicates the predictive power and quality of the model based on cross-validation results, and it should be greater than zero (Uygungil-Erdogan et al., 2025). When Table 6 is examined, it is observed that both R^2^ and Q^2^ values exceed the threshold value, indicating that the model possesses predictive power (0 < R^2^ < 1). The model explains part of the variance in the dependent variable, Q^2^ > 0; the model has predictive power. The findings obtained from the analyses are discussed in more detail in the results section and compared with the literature.

According to the structural model results, the green transformational leadership (GTL) variable explains 35.9% of the variance in the psychological green climate (R^2^ = 0.359). This ratio is considered to be a moderate level of explanatory power in the organizational behavior literature (Cohen et al., 2007). Accordingly, the analysis findings show that GTL significantly strengthens employees’ perceptions of environmental values within the organization. Furthermore, together with GTL and psychological green climate, it explains 51% of the variance in organizational green innovation (R^2^ = 0.510), revealing that leadership and climate factors have a high level of influence on the adoption of innovative environmental practices. Similarly, GTL and psychological green climate together explain 46.5% of the variance in the individual environmental awareness variable (R^2^ = 0.465). This value shows that employees’ environmentally conscious attitudes are strengthened through both leadership guidance and their perceived green climate. Furthermore, the fact that all Q^2^ values (ranging from 0.203 to 0.326) are greater than zero proves that the model has predictive relevance (Hair et al., 2019). These findings show that the model has strong explanatory power overall and that the effects of GTL work meaningfully at both cognitive (climate) and behavioral (innovation and awareness) levels.

## 6 Discussion

### 6.1 Practical/managerial implications

The findings show that leaders’ behaviors, particularly in SMEs, such as environmental vision, role modeling, and motivational guidance, strengthen employees’ environmental awareness and participation in innovative practices. These results reveal that, from a managerial perspective, green leadership behaviors are directly related to organizational performance and sustainability goals, thereby providing a managerial perspective for the design of leadership development, employee awareness, and green innovation training programs.

The first hypothesis determined that GTL has a significant and positive effect on the psychological green climate. The organizational climate, which is shaped by the processes and procedures established by leaders, significantly impacts employees’ perceptions and interpretations (Younis and Hussain, 2023). As a result of leaders incorporating environmental values into an organizational culture and encouraging employees to adopt them, employees often feel psychologically uncomfortable when they engage in activities that contradict these values (Kim et al., 2020). It is considered that the result obtained by assuming that employees’ shared perceptions of organizational processes guide their behavior at work is influenced by organizational climate theory. The results are consistent with those of previous studies in the literature (Zhou et al., 2018). From this perspective, the study highlights the importance of the leader’s role in training and guiding subordinates.

It is expected that the influence of the organizational climate will support employees in developing innovative and environmentally friendly ideas within the framework of the dynamic capabilities theory (Singh et al., 2020). Based on this, the second hypothesis determined that the psychological green climate has a significant and positive effect on organizational green innovation. The results obtained are consistent with both theoretical foundations and the literature (Naz et al., 2023). In addition, the results obtained highlight the importance of creating an appropriate environment or climate within the organization for innovation development.

The inspiring motivations and intellectual encouragement of GTL on environmental issues help employees contribute more to green innovation processes and generate creative solutions for environmental improvements (Graves et al., 2019). From this perspective, the third hypothesis shows that GTL has a significant and positive effect on organizational green innovation. The results of the study are consistent with the literature (Singh et al., 2020; Begum et al., 2022). It can be stated that the study has once again confirmed that employees can act based on the leader’s views, develop an interest in green issues, and transform this interest into innovative products.

In organizations with a high psychological green climate, employees’ sensitivity to environmental issues, both at the organizational level and at the individual level, is rooted in a significant psychological phenomenon based on Organizational Climate Theory (Biswas et al., 2021). Based on this, the fourth hypothesis established in the study demonstrates that the psychological green climate has a significant and positive effect on individual environmental awareness. When there is a strong emphasis on the environment within an organization, employees are more likely to make environmentally conscious decisions not only at work but also in their daily lives (Paillé and Boiral, 2013). No previous study has evaluated these two concepts together in the literature. In this regard, it can be stated that the present study makes a significant contribution to the existing literature.

GTL instills environmental consciousness in its employees through exemplary behavior, contributing significantly to the development of individual environmental awareness (Graves et al., 2019). Based on this, the fifth hypothesis, which was formulated, proves that GTL has a meaningful and positive effect on individual environmental awareness. It is thought that Schwartz’s Norm Activation Model (Schwartz, 1977) has an effect on the results obtained. The study supports the notion that individuals become aware of environmental issues as a result of their environmental behaviors being shaped by ethical and moral norms (Park and Ha, 2014). No previous study has evaluated these two concepts together in the literature. The present study appears to make a modest practical contribution to the literature.

The findings obtained in this study are consistent with previous studies, which support the positive impact of GTL on organizational green innovation. Indeed, Achmada et al. (2024) stated that green leadership practices strengthen organizational sustainability and increase employee participation in green innovation processes. Similarly, Ding et al. (2023) also found that research on green leaders revealed that they improve organizational environmental performance by increasing employees’ environmental awareness and innovation capacity (Ding et al., 2023). These findings support the results of the current study and confirm the impact of green leadership on sustainable business practices.

### 6.2 Theoretical implications

The current study makes several meaningful contributions to the theory. First, the green transformational leader addresses the needs of all stakeholders in line with green, sustainability, and environmental values. As a response to emerging environmental movements, organizations are developing appropriate situational reflexes. In this regard, findings indicate that green innovation increases for the benefit of stakeholders and provides a competitive advantage for the business through the leader’s guidance of employees. The results obtained in this regard point to the effectiveness of stakeholder theory and RBV theories on the concept of green transformational leadership.

Secondarily, the study contributes to the literature theoretically by explaining the importance of leadership in the formation of individual environmental awareness and the underlying factors. It is expected that employees who perform certain actions within the organization will eventually internalize these actions individually and make them part of their lives. As explained by the Norm Activation Model in particular, individuals need suitable role models and environments to shape their environmental behaviors based on moral norms. In this respect, leaders are important figures in the organization and act as a driving force in the formation of individual environmental awareness.

Thirdly, the study expands the GTL literature by addressing how leadership affects not only employee attitudes but also organizational innovation processes and individual environmental awareness within a holistic model. By demonstrating that the effect of GTL occurs at both the organizational and individual levels through the psychological green climate, the study provides a multi-level perspective on leadership theory. In this respect, the study repositioned GTL as a theoretical extension of transformational leadership in the context of environmental sustainability.

### 6.3 Political implications

The research was conducted on SME employees in three different regions. Considering the place of SMEs in Turkey’s employment structure, the research results take on even greater significance. Indeed, small and medium-sized enterprises (SMEs) constitute the vast majority of the business structure in Turkey. According to Turkstat (2024) data, SMEs account for 99.7% of all businesses, 71.4% of employment, 54.2% of wages and salaries, and 64.1% of total turnover. These indicators show that SMEs play a fundamental role in the Turkish economy in terms of both production and employment, and are therefore critical to the success of sustainability and green transformation policies (Turkstat, 2024). The study shows that sustainable development policies in developing economies such as Turkey require not only technological transformation but also transformation based on leadership and organizational culture. The findings indicate that public institutions, development agencies, and support mechanisms such as KOSGEB should prioritize training, incentive, and guidance programs aimed at strengthening green leadership capacity in SMEs. Thus, the research provides practical guidance on the policy-level applicability of green transformation strategies.

### 6.4 Limitations and recommendations for future research

The limited scope of the study, conducted in a restricted area, and the fact that data were collected only from SMEs through a simple sampling method, impose certain limitations on the generalizability of the findings. Additionally, the mood and response style of the participants at the time of answering may also be considered a limitation. However, it should be noted that the results obtained may not occur at the same level in every organizational and cultural context. Indeed, in organizations with highly hierarchical structures, employees participate less in decision-making processes, so the leader’s green value-based guidance may have a limited impact on employee behavior (Kim et al., 2020). Similarly, in cultures with high power distance or authoritarian cultures, even if employees accept the leader’s environmental vision without question, this may prevent the formation of a genuine green climate perception (Hofstede, 2001). In contrast, in participatory cultures and low-hierarchy organizations, the leader’s green-oriented behaviors align more strongly with employees’ voluntary eco-friendly actions (Mittal and Dhar, 2016). These findings suggest that the effect of GTL can be moderated by organizational structure and cultural factors. Therefore, it is recommended that future research consider these contextual variables when explaining the effectiveness of GTL.

In addition, the findings of this study indicate that concrete management tools and programs need to be developed to enable businesses to implement the GTL approach. In this context, it is believed that developing training and certification programs for SME managers could effectively create synergy by combining environmental sustainability principles with transformational leadership skills. It is also recommended that sustainability-focused performance indicators (Green KPIs) be defined (energy savings rate, waste reduction percentage, employee green awareness scores, etc.) to enable businesses to link green innovation and environmentally friendly behaviors to corporate goals. Furthermore, establishing internal mentoring systems where environmentally conscious leaders and employees in SMEs can share experiences with other teams is recommended for its potential to yield effective results. Based on the literature review conducted within the scope of this study, it is recommended that future studies examine the concepts of artificial intelligence, digital transformation, and Industry 5.0 in relation to the current variables.

## 7 Conclusion

This study examined the effect of GTL on organizational green innovation and individual environmental awareness, and evaluated the mediating role of psychological green climate in this relationship. The findings reveal that GTL promotes environmental sustainability practices at both organizational and individual levels. Accordingly, leaders’ visionary approaches that support environmental sustainability increase employees’ environmental awareness and ensure the adoption of green innovation processes within the organization. In addition, a strong psychological green climate within the organization enables employees to participate in environmental sustainability-oriented initiatives with higher motivation and to support green innovation activities.

## Data Availability

The original contributions presented in the study are included in the article/Supplementary material, further inquiries can be directed to the corresponding author.
